# The Differential Effects of Mindfulness and Distraction on Affect and Body Satisfaction Following Food Consumption

**DOI:** 10.3389/fpsyg.2017.01696

**Published:** 2017-09-27

**Authors:** Alice Tsai, Elizabeth K. Hughes, Matthew Fuller-Tyszkiewicz, Kimberly Buck, Isabel Krug

**Affiliations:** ^1^Melbourne School of Psychological Sciences, The University of Melbourne, Melbourne, VIC, Australia; ^2^Murdoch Children’s Research Institute, The Royal Children’s Hospital, Melbourne, Melbourne, VIC, Australia; ^3^Department of Paediatrics, The University of Melbourne, The Royal Children’s Hospital, Melbourne, Melbourne, VIC, Australia; ^4^School of Psychology, Deakin University, Geelong, VIC, Australia; ^5^Centre for Social and Early Emotional Development, Deakin University, Burwood, VIC, Australia; ^6^Southern Synergy, Department of Psychiatry, Monash University, Melbourne, VIC, Australia

**Keywords:** eating pathology, mindfulness, distraction, food consumption, experimental intervention

## Abstract

This study investigated whether engaging in mindfulness following food consumption produced changes in affect and body satisfaction, as compared to a control distraction task. The moderating effects of eating pathology and neuroticism were also examined. A total of 110 female university students consumed food and water before engaging in either a mindfulness induction or a control distraction task. Participants completed trait measures of eating pathology and neuroticism at baseline, and measures of state affect and body satisfaction before and after food consumption, and after the induction. Results revealed that consuming food and water reduced positive affect. Unexpectedly, both the mindfulness group and distraction control group experienced similar improvements in negative affect and body satisfaction following the induction. Eating pathology and neuroticism did not moderate the observed changes. These findings suggest that both mindfulness and distraction may contribute to the effectiveness of treatments for disordered eating that incorporate both of these techniques, such as Dialectical Behavior Therapy.

## Introduction

Mindfulness-based interventions are being increasingly applied to the treatment of eating-related problems in both clinical ED groups (e.g., Wanden-Berghe et al., 2010; Pinto-Gouveia et al., 2017) and non-clinical populations (e.g., Katterman et al., 2014). Mindfulness interventions promote non-judgmental observation and acceptance of one’s experience in the present moment (Kabat-Zinn, 1990), and are often delivered within broader therapeutic frameworks such as ACT (Hayes et al., 1999) and DBT (Linehan, 1993). Despite promising preliminary results for the effectiveness of these therapies (Wanden-Berghe et al., 2010), experimental research delineating the unique contribution of mindfulness to these treatments is lacking. Further, little is known about the immediate impact of mindfulness in the context of disordered eating relevant stressors (e.g., food consumption), or in relation to affective and cognitive outcomes of specific relevance to eating pathology (e.g., body satisfaction). It is also unclear from previous research whether the effectiveness of mindfulness interventions for problematic eating behavior is impacted by factors such as individual differences in eating pathology or personality.

Mindfulness-based interventions are thought to alleviate eating pathology through their effect on maladaptive cognitive and affective processes. Cognitive-behavioral models propose that dysfunctional cognitions related to the importance of weight and shape to self-worth can lead to disordered eating behaviors, such as restricted eating and purging (e.g., Fairburn et al., 2003), which may develop into full threshold EDs if left untreated. Furthermore, eating pathology is associated with experiential avoidance, or the unwillingness to experience unwanted thoughts, emotions, and physical sensations, and active attempts to avoid or suppress them (Hayes et al., 1996). By promoting acceptance of all emotions, thoughts, and sensations, including those that are aversive or unhelpful (Hayes and Feldman, 2004), mindfulness techniques are thought to assist individuals with eating pathology to recognize that thoughts and emotions are temporary, not harmful, and do not necessarily require a reaction or behavioral response (Baer et al., 2005).

In support of this, therapies that incorporate mindfulness-based techniques, such as ACT and DBT, have been shown to reduce ED symptomatology in patients with anorexia nervosa, bulimia nervosa (Hill et al., 2011; Juarascio et al., 2013) and binge ED (Ruffault et al., 2016; Pinto-Gouveia et al., 2017). However, these and other therapies differ widely, with some focusing primarily on mindfulness practice and meditation, and others integrating mindfulness within a broader therapeutic framework (Keng et al., 2011). Given these variations, it is important to isolate the unique contribution of mindfulness to the effectiveness of mindfulness-based treatments for eating-related problems. Preliminary evidence has come from research investigating therapies that are solely or primarily focused on mindfulness training, such as MBSR (Kabat-Zinn, 1990), MBCT (Segal et al., 2002), and MB-EAT (Kristeller and Hallett, 1999; Kristeller and Wolever, 2010). MB-EAT was designed specifically to treat EDs, and has been shown to decrease binge eating frequency and severity in individuals with binge ED (Kristeller and Hallett, 1999; Kristeller et al., 2014). A novel intervention combining elements of MBSR, MBCT, and MB-EAT has also been shown to reduce anxiety and eating in reaction to food-related stimuli in a non-clinical sample of obese women (Daubenmier et al., 2011). Indeed, a recent systematic review found that treatments in which mindfulness meditation was the primary intervention were effective at reducing binge eating and emotional eating in both clinical and non-clinical samples (Katterman et al., 2014).

Although existing research on mindfulness-based therapies for eating pathology is encouraging, to date, only a handful of experimental studies have examined the immediate effects of using mindfulness when faced with potential stressors relevant to disordered eating on outcome variables such as affect and body satisfaction. Stressor tasks utilized in previous studies have included comparing one’s own body to images of thin women in magazines (Wade et al., 2009; Atkinson and Wade, 2012; Svaldi and Naumann, 2014), and eating real food (Cowdrey et al., 2013; Marek et al., 2013). Overall, the pattern of findings suggests a differential effect depending on clinical status, with mindfulness having neutral or negative effects on affect and body satisfaction in clinical samples (Marek et al., 2013; Svaldi and Naumann, 2014), and a neutral or positive effect in non-clinical samples (Wade et al., 2009; Adams et al., 2012; Atkinson and Wade, 2012). Most strikingly, Marek et al. (2013) found that a mindful eating exercise actually increased negative affect in participants with diagnosed EDs.

Despite the growth of experimental research in this area, a number of questions remain unanswered. Only one study has examined the effect of mindfulness on positive affect, with no significant changes reported in either individuals with EDs or in healthy controls (Marek et al., 2013). Further research is required to verify these findings. Importantly, only two studies have investigated mindfulness inductions in relation to food consumption, and both studies utilized mindfulness *prior* to food being consumed (Cowdrey et al., 2013; Marek et al., 2013). Even in non-clinical groups, food consumption has been shown to produce immediate and adverse effects on body image (e.g., Hayes et al., 2011) and estimations of body size (e.g., Thompson et al., 1993). Although a mindfulness induction prior to food consumption could help to prevent these responses or help individuals cope with them as they occur, it is possible that mindfulness inductions might have greater impact when introduced *after* food consumption to help individuals cope with aversive thoughts or feelings that arise after eating.

The primary aim of the study was to investigate how engaging in a mindfulness induction task after food consumption impacted affect and body satisfaction in female university students, as compared to a control distraction task. Distraction, or the act of moving one’s attention away from distressing thoughts or activities toward more pleasant or emotionally neutral thoughts or tasks (Nolen-Hoeksema and Morrow, 1991), was chosen as a control condition as it could be considered to be the opposing state of being in relation to mindfulness (Marek et al., 2013) and has been employed as a control task in a number of previous studies (Cowdrey et al., 2013; Marek et al., 2013; Arch et al., 2016). The items selected for food consumption were a chocolate muffin and a cup of water. In line with Marek et al. (2013), who operationalized food exposure by providing participants with a portion of cake, the food item selected for this study was intended to be desirable yet unhealthy, and thus function as a potential stressor relevant to disordered eating. A cup of water was provided to increase the physical sensation of satiation. Based on prior evidence that the consumption of foods perceived to be unhealthy is related to post-consumption increases in negative mood (e.g., Macht and Dettmer, 2006) and can also produce immediate negative effects on body satisfaction (e.g., Hayes et al., 2011) and estimations of body size (e.g., Thompson et al., 1993), it was expected that the consumption of these items in the current study would have a negative impact on affect and body satisfaction.

A secondary aim was to explore whether two individual difference factors known to be related to problematic eating behaviors, namely, eating pathology and neuroticism, moderated the effect of the mindfulness induction on outcome variables and/or acted as predictors of change after food consumption. The apparent differential effects of mindfulness on affect and body satisfaction for clinical and non-clinical groups described above (e.g., Atkinson and Wade, 2012; Marek et al., 2013) suggest that eating pathology severity may influence the effectiveness of mindfulness techniques in the context of disordered eating relevant stressors. For example, it is possible that individuals with more severe eating pathology find mindfulness exercises distressing, perhaps due to these exercises heightening awareness of difficult emotions, body dissatisfaction, or unpleasant physical sensations (e.g., hunger, fullness) (Cowdrey et al., 2013; Marek et al., 2013). Neuroticism, a broad personality dimension reflecting individual differences in emotional stability and the tendency to experience negative affect (DeNeve and Cooper, 1998), is consistently and positively associated with disordered eating behaviors and cognitions (Cassin and von Ranson, 2005) and negatively associated with trait mindfulness (Giluk, 2009). However, no experimental studies have examined the possible influence of neuroticism in the context of mindfulness interventions for disordered eating. Assessing the potential influence of these trait factors is important because if the effect of mindfulness changes according to level of eating pathology or neuroticism, mindfulness-based interventions may benefit some individuals more than others.

The study was designed to address a number of the limitations and gaps in previous research by inducing mindfulness after food consumption (rather than prior to food consumption), measuring the impact of mindfulness on both negative and positive affect, and testing the moderating effects of neuroticism and eating pathology. It was hypothesized that: (1) negative affect would increase and positive affect and body satisfaction would decrease from pre- to post-food consumption in the overall sample; (2) greater levels of eating pathology and neuroticism would be associated with a greater increase in negative affect and decrease in positive affect and body satisfaction from pre- to post-food consumption; (3) the mindfulness group would report greater increases in state mindfulness, positive affect and body satisfaction and greater reduction in negative affect from pre- to post-induction than the distraction group; and (4) eating pathology and neuroticism would moderate the effect of the mindfulness induction. Given the lack of available literature on the role of these individual factors in relation to mindfulness interventions, no hypotheses were made regarding the direction of these moderation effects.

## Materials and Methods

### Participants

Participants were 110 (aged 18–42 years, *M* = 19.30, *SD* = 2.50) female university students who took part in return for course credit. Participant BMIs ranged from 16.7 to 32.1 (*M* = 21.44, *SD* = 2.91), with 70.9% in the normal/healthy weight range, 18.2% classified as underweight, 9.1% as overweight and 1.8% obese. All participants provided informed consent prior to completing the study. Ethics approval was gained from a university in Melbourne.

### Materials

#### Demographics

Participants provided their date of birth, age, ethnicity, country of birth, years lived in Australia, education, employment status, marital status, and current height and weight. BMI was calculated based on self-reported height and weight.

#### Negative and Positive Affect

The PANAS (Watson et al., 1988) was used to measure state positive and negative affect at three time points throughout the experiment: pre-food consumption (T1), post-food consumption (T2), and post-induction (T3). The PANAS comprises a list of 20 emotions, 10 of positive valence (e.g., “Excited”), and 10 of negative valence (e.g., “Upset”). Participants indicated the extent to which they felt each emotion at the present moment on a 5-point Likert scale (1 = *Very slightly or not at all*; 5 = *Extremely*). Total scores for positive and negative affect were summed separately. The PANAS has previously demonstrated good internal consistency reliability, test–retest reliability, convergent validity, and discriminant validity (Watson et al., 1988; Watson and Clark, 1994). In the current study, Cronbach’s alphas at the three time points were 0.88 (T1), 0.91 (T2), and 0.92 (T3) for positive affect and 0.86 (T1), 0.83 (T2), and 0.87 (T3) for negative affect.

#### Body Satisfaction

Body satisfaction was measured at all three time points using the BISS (Cash et al., 2002). The BISS is a 6-item scale assessing a person’s evaluation of, and feelings about their body and physical appearance in the present moment. For all items, participants were instructed to select the statement that best described how they felt “right now at this very moment” on a 9-point Likert scale (e.g., “*Right now I feel…* ‘*Extremely dissatisfied* with my weight’ = 1; ‘*Extremely satisfied* with my weight’ = 9”). Lower mean scores indicated more negative body satisfaction. The BISS has previously been found to have acceptable internal consistency and convergent validity, and be moderately stable with a test–retest reliability of *r* = 0.69 over 2–3 weeks (Cash et al., 2002). Cronbach’s alphas in the current study range from 0.74 (T1) to 0.79 (T3).

#### State Mindfulness

The 13-item TMS (Lau et al., 2006) was used to measure state mindfulness at post-food consumption (T2) and post-induction (T3). The TMS consists of two subscales: *curiosity* (6 items), which assesses the extent to which one approaches the present moment with an attitude of curiosity (e.g., “I remained curious about the nature of each experience as it arose”), and *de-centering* (7 items), which assesses one’s ability to attend to the present moment with a sense of detachment (e.g., “I experienced myself as separate from my changing thoughts and feelings”). Participants indicated the extent to which each statement described their experience on a 5-point scale (4 = *Very much;* 0 = *Not at all*). Total scores were computed for each sub-scale, with higher scores indicating higher levels of each aspect of state mindfulness. The TMS has exhibited good internal consistency (α = 0.88 for the curiosity subscale and α = 0.84 for the decentering subscale), as well as acceptable convergent and discriminant validity with other measures (Lau et al., 2006). In the current study, Cronbach’s alphas ranged from 0.82 (T3, curiosity) to 0.93 (T3, de-centering).

#### Eating Pathology

The EAT-26 (Garner et al., 1982) was administered at T1 to measure eating pathology. The EAT-26 comprises 26 items assessing a variety of current disordered eating symptoms (e.g., “I am occupied with a desire to be thinner”). Participants indicated the frequency with which each statement applied to them on a 6-point scale (3 = *Always*, 2 = *Usually*, 1 = *Often*, 0 = *Sometimes*, 0 = *Rarely*, 0 = *Never*). Scores were summed, with total scores of 20 or above indicating a high level of concern regarding problematic eating behavior, body weight, or dieting. The EAT-26 has demonstrated good internal consistency (α = 0.83–0.90) and test–retest reliability (*r* = 0.84) in previous samples of young women (Garner et al., 1982; Carter and Moss, 1984), as well as good convergent validity with BMI and the body dissatisfaction and drive for thinness subscales of the Eating Disorders Inventory-2 (Doninger et al., 2005). Cronbach’s alpha for the EAT-26 in the present study was 0.82.

#### Neuroticism

Neuroticism was assessed at T1 using the 20-item neuroticism scale of the IPIP (Goldberg, 1999). The scale includes 10 negatively keyed items (e.g., “Am filled with doubts about things”) and 10 positively keyed items (e.g., “Am relaxed most of the time”). Participants indicated how accurately each statement described them using a 5-point Likert scale (1 = *Very inaccurate*; 5 = *Very accurate*). Scores were summed, with higher scores indicating higher levels of neuroticism. The IPIP has relatively high reliability and convergent validity with other measures of personality (Goldberg, 1999). Cronbach’s alpha in the present study was 0.89.

#### Food

A chocolate muffin (115 g) and a cup of water (175 ml) were provided for each participant to consume following the T1 questionnaires. Water was provided to maximize the effect of food consumption by increasing the physical sensation of fullness. Remaining portions of the muffin and water were later measured to calculate the amount of food and water consumed by each participant.

#### Experimental Conditions

Both experimental tasks were viewed on a full-screen video on a computer and featured a series of 45 statements appearing in white text in the center of a blue background. Each statement was displayed for 13.33 s and was accompanied by a corresponding voiceover (male speaker) played through headphones. The videos were 10 min in length and were hosted on YouTube and embedded into the survey on Qualtrics. The full scripts used in each condition can be obtained from the corresponding author on request.

#### Mindfulness Induction

The content of the mindfulness induction consisted of the “Practicing Awareness of Your Experience” exercise drawn from the original ACT manual (Hayes et al., 1999, p. 179), with minor edits to divide the continuous script into 45 individual items. The induction progressively led participants through a brief mindfulness exercise in which they were guided to become aware of their body (e.g., “Become aware of the physical position of your arms”), breath (e.g., “Become aware of your breathing”; “Follow a breath as it comes in through your nose”), and any thoughts, feelings, or sensations they experienced with non-judgmental awareness (e.g., “As thoughts come into your awareness, just watch them”).

#### Control Distraction Task

The content of the distraction task was adapted from Nolen-Hoeksema and Morrow (1993), and involved attending to 45 statements that were externally oriented and unrelated to the self. Some items were modified to make them more familiar for Australian participants (e.g., “Think about and picture the UCR watch tower” was replaced with “Think about and picture the Sydney Harbor Bridge”). Other items were modified to the modern context (e.g., “Think about the parking lot at a drive-in” was replaced with “Think about the parking lot at a cinema”).

### Procedure

**FIGURE 1:**
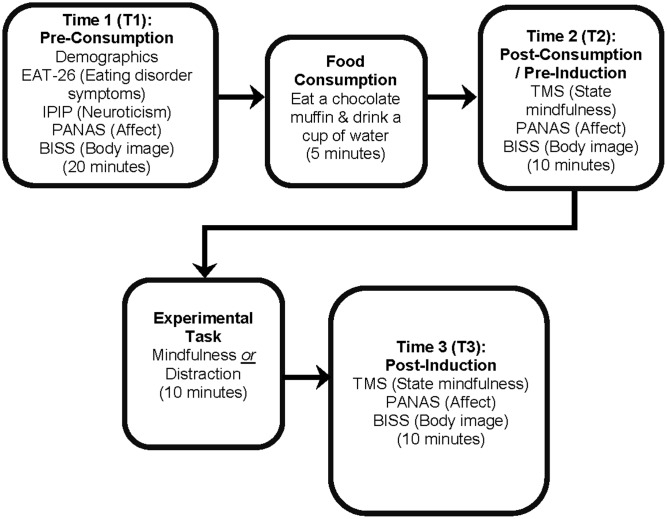
The experimental procedure. Each phase of the experiment is in bold, with questionnaire measures or experimental tasks displayed underneath. The approximate duration of each phase is presented at the bottom of each phase in parentheses.

The procedure is depicted in **Figure 1**. At the first time point (T1), participants completed baseline questionnaires assessing demographics, trait-level eating pathology and neuroticism, and state affect and body satisfaction. Participants were then provided with a chocolate muffin and a cup of water to consume. On-screen instructions asked participants not to engage in any other activity while eating, and informed them that they could leave any unfinished food and water next to the computer. At the second time point (T2), after each participant had finished eating, the experimenter removed the remaining food and water and progressed the survey to the next section for participants to complete the mindfulness measure and repeat the state measures of affect and body satisfaction. Next, participants were randomly assigned to one of the two experimental conditions using the “Randomizer” function on the web-based survey software Qualtrics (Qualtrics, 2015) and completed either the mindfulness induction or control distraction task. At the third time point (T3), following the experimental manipulation, participants again completed the measures of state mindfulness, affect, and body satisfaction. The total duration of the experiment was approximately 1 h.

## Results

### Descriptive Statistics

**Table 1** presents summary statistics for all demographic variables for the overall sample by experimental group (mindfulness vs. distraction). Chi-square and *t*-test analyses confirmed that the two groups did not differ significantly on demographic variables. The majority of participants (71%) fell within the healthy BMI range of 18.50–24.99.

**Table 1:** Demographics of the sample, the mindfulness and distraction groups, and Chi-square statistics.

Demographics		Total sample (*N* = 110)		Mindfulness group (*n* = 54)		Distraction group (*n* = 56)		Test statistics		
					
		*n*	*%*	*n*	*%*	*n*	*%*	χ^2^	*df*	*p*
Ethnicity^a^	Caucasian	44	40	21	39	23	41	1.69	2	0.431
	Asian	54	49	29	54	25	45			
	Other^1^	12	11	4	7	8	14			
Country of birth	Australia	60	55	25	46	35	63	2.91	1	0.088
	Other	50	45	29	54	21	37			
Years in Australia	≤2 years	29	58	16	55	13	62	0.23	1	0.634
(if born overseas)	≥3 years	21	42	13	45	8	38			
Education^a^	Secondary school	77	70	40	74	37	70	1.58	2	0.454
	Some tertiary	32	29	14	26	18	29			
	University degree^2^	1	1	0	0	1	1			
Employment^a^	Student	76	69	37	69	39	70	1.15	3	0.764
	Part-time	17	16	8	15	9	15			
	Casual	13	12	6	11	7	13			
	Unemployed	4	4	3	6	1	2			
Marital status^a^	Single	87	79	40	74	47	84	1.61	1	0.204
	In a relationship	23	21	14	26	9	16			
		*M*	*SD*	*M*	*SD*	*M*	*SD*	*t*	*df*	*p*
	
Age		19.30	2.50	19.63	3.37	18.98	1.09	1.37	108	0.175
BMI		21.44	2.91	21.65	3.15	21.24	2.67	0.73	108	0.469


*^a^Categories on these measures were collapsed and/or removed due to low cell counts. ^1^“Other” combined a minority of participants who identified as African American or Black, Hispanic or Latin American, Middle Eastern, Native Hawaiian or Other Pacific Islander, or Other. ^2^University degree or equivalent.*

**Table 2** presents summary statistics for all key variables of interest at T1 by experimental group. There were no significant differences between the groups. Scores on the EAT-26 were low, with 88% of participants scoring below the clinical threshold (≥20) for a possible diagnosis of an ED. Mean positive affect scores were higher than negative affect scores, and mean scores for body image indicated moderate levels of body satisfaction across both groups.

**Table 2:** Comparisons between the mindfulness and distraction groups on key variables at T1.

	Experimental condition						
	
	Mindfulness (*n* = 54)			Distraction (*n* = 56)			Test statistics	
			
	*M*	*SD*	Range	*M*	*SD*	Range	*t*	*p*
Eating pathology	10.19	7.91	0–32	8.09	7.93	0–35	1.39	0.168
Neuroticism	58.13	11.96	35–86	56.93	12.51	33–84	0.51	0.608
Negative affect	14.26	5.68	10–38	13.80	4.78	10–37	0.46	0.650
Positive affect	23.92	7.68	11–44	25.98	8.24	13–49	0.02	0.981
Body image	4.96	1.20	2.17–7.83	5.08	1.18	3–7.17	-0.52	0.606
Mindfulness – curiosity	12.11	6.22	0–24	13.11	6.03	0–24	-0.85	0.395
Mindfulness – de-centering	12.26	4.96	0–23	13.38	5.44	2–26	-1.12	0.264
Muffin consumed (g)	88.00	31.67	8–115	89.96	28.39	32–115	-0.34	0.732
Water consumed (ml)	157.67	38.75	20–175	163.70	32.12	13–175	-0.89	0.376


*g, grams; ml, milliliters.*

### Post-food Consumption Changes (Hypotheses 1 and 2)

A series of paired sample t-tests revealed significant decreases in positive affect from T1 to T2; *t*(109) = 2.96, *p* = 0.004 (two-tailed). Changes in negative affect [*t*(109) = 0.99, *p* = 0.322] and body satisfaction [*t*(109) = 1.42, *p* = 0.158] were non-significant.

Hierarchical regressions (controlling for T1 scores on the dependent variables) were undertaken to evaluate predictors of affect and body satisfaction at T2 (i.e., after food consumption). Amount of food consumed and BMI were included as potential covariates, given their established association with mood and body satisfaction (Macht, 2008; Weinberger et al., 2016). As shown in **Table 3**, negative affect scores at T2 were higher for individuals with more severe eating pathology. None of the other proposed predictors were significantly associated with T2 affect or body satisfaction. Moreover, their collective incremental improvement in prediction (after controlling for affect and body satisfaction at T1) was small and non-significant (Δ*R*^2^ ranged from 0.002 to 0.054 for the three models).

**Table 3:** Hierarchical regression predicting affect and body image following muffin consumption.

Dependent Variable		Δ*R*^2^	Predictors	*B*	*SE*	*b*	*sr*	*t*	*p*
Negative affect (T2)	Step 1	0.333^∗∗^							<0.001
			Negative affect (T1)	0.49	0.07	0.58	0.58	7.35	<0.001
	Step 2	0.054							0.065
			Negative affect (T1)	0.41	0.08	0.48	0.41	5.30	<0.001
			Eating pathology	0.13	0.05	0.22	0.20	2.65	0.009
			Neuroticism	0.03	0.03	0.07	0.06	0.81	0.423
			Body mass index	-0.08	0.12	-0.05	-0.05	-0.69	0.490
			Muffin consumption	0.00	0.01	0.01	0.02	0.17	0.868
Positive affect (T2)	Step 1	0.678^∗∗^							<0.001
			Positive affect (T1)	0.91	0.06	0.82	0.82	15.10	<0.001
	Step 2	0.002							0.945
			Positive affect (T1)	0.92	0.06	0.83	0.81	14.67	<0.001
			Eating pathology	0.00	0.07	0.00	0.00	-0.05	0.957
			Neuroticism	0.01	0.04	0.01	0.01	0.18	0.859
			Body mass index	-0.10	0.17	-0.03	-0.03	-0.59	0.555
			Muffin consumption	0.01	0.02	0.03	0.05	0.52	0.606
Body satisfaction (T2)	Step 1	0.571^∗∗^							<0.001
			Body satisfaction (T1)	0.75	0.06	0.76	0.76	11.98	<0.001
	Step 2	0.016							0.424
			Body satisfaction (T1)	0.69	0.08	0.69	0.55	8.72	<0.001
			Eating pathology	-0.01	0.01	-0.08	-0.07	-1.10	0.275
			Neuroticism	-0.01	0.01	-0.07	-0.06	-0.96	0.339
			Body mass index	-0.02	0.03	-0.04	-0.04	-0.62	0.536
			Muffin consumption	0.00	0.00	-0.08	-0.08	-1.27	0.209


**B*, Unstandardized coefficients; *SE*, Standard error; *b*, Standardized coefficients; *sr*, semipartial correlation. ^∗∗^*p* < 0.001.*

### Post-induction Changes and Moderators (Hypotheses 3 and 4)

Two-way ANOVAs (group: mindfulness vs. distraction; time: T2 vs. T3) were undertaken to evaluate the impact of the inductions on study variables. Time effects were observed for all variables except positive affect. Negative affect significantly decreased [*F*(1,108) = 29.34, *p* < 0.001, $\eta_{p}^{2}$ = 0.21], whereas body satisfaction [*F*(1,108) = 14.46, *p* < 0.001, $\eta_{p}^{2}$ = 0.12], curiosity [*F*(1,108) = 20.95, *p* < 0.001, $\eta_{p}^{2}$ = 0.16], and de-centering [*F*(1,108) = 58.55, *p* < 0.001, $\eta_{p}^{2}$ = 0.35], all increased post-induction. However, time by group interactions were non-significant for all variables (*ps* > 0.469, $\eta_{p}^{2}$ < 0.01).

Hierarchical regressions (controlling for T2 scores on the dependent variables) were undertaken to evaluate predictors and moderators of affect and body satisfaction at T3. All independent variables were mean-centered to reduce non-essential collinearity. For the model predicting negative affect at T3, Step I (with only T2 negative affect) accounted for 63% variance in the dependent variable; *F*(1,108) = 179.71, *p* < 0.001. Inclusions of group (mindfulness vs. distraction), eating pathology, neuroticism, BMI, and food consumption at Step II did not significantly improve the model; Δ*R*^2^ = 0.02, *F*(5,103) = 1.25, *p* = 0.290. Similarly, inclusion of interaction terms between group and eating pathology, and group and neuroticism at Step III did not explain significant additional variance; Δ*R*^2^ = 0.01, *F*(2,101) = 0.66, *p* = 0.518. T2 negative affect remained significant at each step.

For the model predicting positive affect at T3, T2 positive affect accounted for 60% of the variance at Step I; *F*(1,108) = 157.61, *p* < 0.001. Inclusion of the remaining variables at Step II [Δ*R*^2^ = 0.01, *F*(5,103) = 0.30, *p* = 0.914], and interaction terms at Step III [Δ*R*^2^ = 0.00, *F*(2,101) = 0.05, *p* = 0.951] did not significantly improve the model. T2 positive affect remained significant at each step.

For the model predicting body satisfaction at T3, body satisfaction scores at T2 accounted for 52% of the variance at Step I; *F*(1,108) = 116.32, *p* < 0.001. Inclusion of the remaining variables at Step II [Δ*R*^2^ = 0.03, *F*(5,103) = 1.15, *p* = 0.337] and the interaction terms entered at Step III [Δ*R*^2^ = 0.01, *F*(2,101) = 0.40, *p* = 0.670] did not significantly improve the overall model. T2 body satisfaction remained significant at each step. In addition, individuals with higher eating pathology had lower body satisfaction ratings at T3. None of the other predictors significantly contributed to the dependent variable.

For brevity, only Step III results for each of these models are presented in **Table 4**. Results of earlier steps are available upon request.

**Table 4:** Results of third step of hierarchical regression for affect and body satisfaction.

Dependent Variable	Predictors	*B*	*SE*	*b*	*sr*	*t*	*p*
Negative affect (T3)	Negative affect (T2)	0.66	0.06	0.75	0.66	11.19	<0.001
	Body mass index	-0.14	0.08	-0.11	-0.11	-1.78	0.078
	Muffin consumed	0.01	0.01	0.08	0.08	1.28	0.203
	Eating pathology	0.03	0.03	0.07	0.06	0.98	0.328
	Neuroticism	0.02	0.02	0.05	0.05	0.82	0.415
	Group	-0.53	0.46	-0.07	-0.07	-1.14	0.258
	Group^∗^Eating pathology	-0.03	0.06	-0.03	-0.03	-0.49	0.623
	Group^∗^Neuroticism	-0.04	0.04	-0.06	-0.05	-0.88	0.382
Positive affect (T3)	Positive affect (T2)	0.79	0.07	0.77	0.75	11.89	<0.001
	Body mass index	-0.03	0.19	-0.01	-0.01	-0.15	0.880
	Muffin consumed	-0.01	0.02	-0.02	-0.02	-0.36	0.722
	Eating pathology	0.07	0.08	0.06	0.06	0.87	0.386
	Neuroticism	0.01	0.05	0.01	0.01	0.13	0.896
	Group	0.41	1.14	0.02	0.02	0.36	0.719
	Group^∗^Eating pathology	-0.04	0.15	-0.02	-0.02	-0.25	0.806
	Group^∗^Neuroticism	-0.01	0.10	-0.01	-0.01	-0.13	0.896
Body Satisfaction (T3)	Body satisfaction (T2)	0.61	0.08	0.65	0.53	7.90	<0.001
	Body mass index	0.02	0.03	0.05	0.05	0.71	0.480
	Muffin consumed	0.00	0.00	0.00	0.01	0.05	0.964
	Eating pathology	-0.02	0.01	-0.16	-0.14	-2.11	0.038
	Neuroticism	0.00	0.01	-0.01	0.01	-0.20	0.845
	Group	0.01	0.15	0.01	0.01	0.07	0.946
	Group^∗^Eating pathology	0.00	0.02	0.01	0.01	0.08	0.937
	Group^∗^Neuroticism	-0.01	0.01	-0.07	-0.06	-0.89	0.377


**B*, Unstandardized coefficients; *SE*, Standard error; *b*, Standardized coefficients; *sr*, semipartial correlation.*

## Discussion

This study focused on the potential of mindfulness to influence affective and cognitive processes involved in disordered eating in young women. As hypothesized, positive affect decreased following food consumption, and although negative affect and body satisfaction remained unchanged overall, participants with more severe eating pathology reported a greater increase in negative affect post-food consumption. Unexpectedly, both the mindfulness induction and the control distraction task were related to a subsequent increase in state mindfulness, as well as increased body satisfaction and decreased negative affect. There was no effect of mindfulness or distraction on positive affect. Finally, neither eating pathology nor neuroticism moderated the observed changes in affect or body satisfaction from pre- to post-induction.

### Effect of Food Consumption on Affect and Body Satisfaction

The finding that negative affect did not increase after food consumption is perhaps unsurprising given that the majority of participants scored below the clinical threshold on the eating pathology measure. However, the finding that greater eating pathology severity predicted higher negative affect after food consumption is in line with previous daily monitoring research indicating that mood was worse on the days with self-reported binge eating episodes than on non-binge days (Schulz and Laessle, 2010; Kukk and Akkermann, 2017). Given that food is a natural and powerful reward, it is not generally expected to trigger strong aversive reactions in healthy individuals (Berridge, 1996). It is perhaps telling then, that positive affect decreased after eating, which is inconsistent with a response of pleasure after reward, and instead suggests that the food consumption task dampened positive mood in this non-clinical sample.

The lack of significant changes in body satisfaction after food consumption contradicts previous research, which has shown that that body image is immediately and adversely affected by food consumption in non-clinical samples (e.g., Vocks et al., 2007; Hayes et al., 2011). However, while the participants in these past studies (Vocks et al., 2007; Hayes et al., 2011) were required to consume the entire food item (e.g., donut, banana, or milkshake), participants in this study were not forced to do so for ethical reasons. Therefore, it is possible that participants ceased consumption when eating began to have an adverse effect on body image satisfaction.

### Effect of Mindfulness on Negative Affect

Our findings are consistent with those from previous experimental studies showing that a mindfulness exercise reduced negative affect in non-clinical groups after exposure to a stressor (Atkinson and Wade, 2012; Marek et al., 2013). Furthermore, these improvements occurred despite negative affect not being significantly impacted by eating. This suggests that mindfulness could be used to regulate negative emotions even when a negative reaction has not been triggered by a disordered eating relevant stressor. In fact, regular practice of mindfulness as an emotion regulation strategy in the absence of negative emotion may prepare individuals to cope adaptively when aversive reactions do occur. The importance of regular mindfulness practice has been emphasized in theory, research, and treatment to increase mindful awareness in everyday life, and thus maximize therapeutic benefits (Carmody and Baer, 2008; Vettese et al., 2009). As negative affect has been found to be a risk factor for the development of eating pathology (Stice, 2002), the reduction of negative affect after a mindfulness exercise in this non-clinical sample highlights its potential value for illness prevention and early intervention.

### Effect of Mindfulness on Positive Affect

The results showed that there were no overall changes in positive affect from pre- to post-induction in the mindfulness group. This mirrors the finding from the sole experimental study that investigated positive affect in this literature (Marek et al., 2013). Theoretically, the aim of mindfulness is not to generate positive mood, as “mindfulness is not about getting anywhere else or fixing anything” (Kabat-Zinn, 2003, p. 148). Instead, it encourages the acceptance of one’s present experience, regardless of its perceived valence (Chambers et al., 2009). It is possible that through such acceptance, the mindfulness induction stabilized positive affect and prevented its further deterioration after food consumption.

### Effect of Mindfulness on Body Satisfaction

As expected, the mindfulness group reported increases in body satisfaction from pre- to post-induction. One possible mechanism underlying this effect may be the attenuation of negative, distorted cognitions and perceptions about body image. As mindfulness involves being open to all experiences as they arise, it does not favor or avoid specific thoughts, feelings, and occurrences over others (Kiken and Shook, 2012). Accordingly, it has been theorized that mindfulness reduces the influence of bias, prejudice, and pre-existing beliefs, facilitating greater clarity and accuracy in perception and cognition (Brown et al., 2007). By diminishing inaccurate cognitions about body image while cultivating non-reactivity to thoughts and emotions (Baer et al., 2005), mindfulness may thus reduce the urge to engage in disordered eating behaviors to counteract thoughts and feelings of body dissatisfaction (Fairburn et al., 2003).

Evidence for this potential mechanism has been shown in an aforementioned study that examined disturbances in body image perception after the *imagined* eating of fattening food (Rawal et al., 2011). In this study, participants who completed a mindfulness exercise before the imagined eating task provided lower estimates of their weight afterwards, compared to those who underwent a self-analytical exercise. Furthermore, those with higher eating pathology in the mindfulness condition reported a lower likelihood that their weight or shape had changed as a result of imagined eating (Rawal et al., 2011), as compared to the analytical condition. This suggests that mindfulness may have helped to correct distorted and negative cognitions about body weight and shape, and enabled individuals to evaluate their body more realistically (Brown et al., 2007). This process may have occurred in the current study, allowing any pre-existing negative body image perceptions to be corrected, even if body satisfaction had not been adversely impacted by food consumption. As body dissatisfaction is “one of the most consistent and robust risk and maintenance factors for eating pathology” (Stice, 2002, p. 833), improving body satisfaction through engaging in mindfulness may be a promising way to mitigate its effects.

### Mindfulness and Distraction: No Group Differences

Perhaps the most intriguing finding was that the mindfulness and distraction groups did not differ on state mindfulness, affect, or body satisfaction from pre- to post-induction. This contradicts some other research (e.g., Arch et al., 2016), but is consistent with research by Wade et al. (2009), which found that mindfulness and distraction were comparable in their effectiveness in improving weight and appearance satisfaction, when compared to a rumination condition.

Although the control distraction task used in the current study involved thinking about items that were unrelated to the self or present experience, participants may have nonetheless engaged in the task in a mindful manner. In fact, mindfulness practice can vary from formal mindfulness meditation through to informal practices that aim to promote constant awareness in all activities of everyday life (Kabat-Zinn, 2003). In this sense, any activity can be considered a mindfulness exercise when approached mindfully. Therefore, the distraction exercise may have involved “turning one’s attention to something else” (Linehan, 2014, p. 440) with purposeful, mindful awareness as opposed to completely suppressing or rejecting experiences in the present moment (Bishop et al., 2004). Consequently, improvements on negative affect and body dissatisfaction in the distraction group may have occurred through mindfulness processes.

Importantly, although distraction has been employed as a control condition in previous research investigating mindfulness-based interventions (e.g., Marek et al., 2013), distraction itself can be used as a therapeutic technique (e.g., Linehan, 1993). It is therefore plausible that the distraction task may have led to improvements in outcomes through processes that were independent of observed increases in mindfulness. In fact, distraction is one of many distress tolerance skills that are taught alongside mindfulness in DBT (Linehan, 1993, 2014). The lack of difference between the mindfulness and distraction groups on outcome variables may indicate that both techniques contribute to the effectiveness of DBT as a treatment for disordered eating, despite its classification as a “mindfulness-based” intervention (e.g., Keng et al., 2011). These findings also suggest that both techniques may be valuable in addressing disordered eating in non-clinical samples, although further research is needed to confirm this (Wade et al., 2009).

### Eating Pathology and Neuroticism: No Evidence of Moderation

Although a substantial minority of participants (12%) demonstrated eating pathology at a level above the threshold for clinical concern [a proportion comparable with prevalence rates of disordered eating in female undergraduates (Luce et al., 2008) and in women in the general population (Solmi et al., 2014)], eating pathology did not moderate improvements in negative affect and body satisfaction from pre- to post-induction. Similarly, neuroticism was also found not to moderate the improvement in negative affect and body satisfaction from pre- to post-induction. Therefore, it appears that mindfulness produced beneficial outcomes regardless of individual differences in eating pathology and neuroticism.

However, it needs to be mentioned that for our regression analyses predicting body dissatisfaction (or rather change post-induction), eating pathology was a significant predictor. This may suggest that regardless of experimental condition, individuals with higher levels of eating pathology had less improvement in body satisfaction, compared to those participants scoring low on the eating pathology measure. The group by eating pathology interaction was not significant, but this might have been because of the type of tasks used – e.g., we might have found that distraction helps individuals with both high and low levels of eating pathology, but mindfulness may only help people with low levels. Since both experimental manipulations seemed to have a similar effect, it is therefore perhaps not surprising that eating pathology was a significant predictor regardless of group. It is also possible that this finding could have just been an effect of time, in that body satisfaction may increase over time regardless of the type of induction or task, but that this happens more slowly or not at all for individuals scoring higher on eating pathology.

### Limitations

This study has a number of limitations that must be acknowledged. First, we only recruited female participants. It would be beneficial for future research to recruit male participants to explore potential gender differences. Second, including other control groups that completed alternative experimental tasks such as no task or rumination (Wade et al., 2009) may have provided informative comparisons and helped to clarify whether the observed changes were due to time alone (i.e., time elapsing since food consumption) or were associated with the content of the inductions. Relatedly, although implementing mindfulness after food consumption was a novel strength of this study, including additional control groups who were administered the mindfulness induction before eating and during eating would have further strengthened the findings and allowed us to determine the optimal time to employ mindfulness techniques in relation to food consumption. Third, participants’ past experience with mindfulness or meditation was not accounted for, and may have impacted their response to the mindfulness exercise and its effect on outcomes. Fourth, the use of a non-clinical university sample reduces the generalizability of findings to other populations, particularly clinical populations. Fifth, it is possible that because prospective participants were informed that the study involved the consumption of food (for ethical reasons), the sample may have been biased toward individuals who were less likely to perceive the food consumption task as a stressor and had lower levels of eating pathology. However, given that a similar proportion of participants demonstrated clinically concerning levels of eating pathology to prevalence rates reported in other non-clinical female samples (Luce et al., 2008; Solmi et al., 2014), the impact of this potential limitation may have been minimal. Sixth, reliance on self-reported weight and height to calculate BMI may have led to an inaccurate or imperfect calculation of BMI scores. A final limitation is that participants were not provided with specific instructions regarding their eating behavior prior to the experiment, nor were any hunger ratings recorded prior to food consumption. These omissions may have impacted the amount of food consumed and the subsequent degree of distress experienced. It would be beneficial for future studies to control for these variables.

### Clinical Implications

The findings of this study suggest that engaging in mindfulness or distraction exercises may help young women to improve negative affect and body satisfaction after eating, irrespective of levels of eating pathology or neuroticism, and regardless of whether eating adversely affected these outcomes. These techniques do not necessarily need to be taught in isolation: they may constitute two strategies in a ‘toolkit’ of techniques that individuals can draw upon to cope adaptively with dysfunctional or unpleasant thoughts and feelings related to eating and body image. The results of this study also point to the potential for mindfulness and distraction techniques to be used in prevention and early intervention programs for young women in the general population, by targeting negative affect and body dissatisfaction as risk and maintenance factors in disordered eating (Stice, 2002; Atkinson and Wade, 2014).

## Conclusion

This study found that engaging in a short mindfulness exercise after eating reduced negative affect and improved body satisfaction in female university students. Interestingly, engaging in a distraction exercise resulted in comparable benefits. This suggests that mindfulness and distraction both contribute to the effectiveness of mindfulness-based interventions that incorporate these techniques (e.g., DBT). Neuroticism and eating pathology did not moderate the effects of the experimental tasks, suggesting that the observed benefits should hold across women who differ on these factors in the non-clinical population. Therefore, teaching mindfulness or distraction exercises to young women may help to attenuate the influence of negative affect and body dissatisfaction as risk factors in the development and maintenance of disordered eating (Stice, 2002), and thus play a role in illness prevention and early intervention.

## Ethics Statement

This study was carried out in accordance with the recommendations of the HESC ethics committee at the University of Melbourne with written informed consent from all subjects. All subjects gave written informed consent in accordance with the Declaration of Helsinki. The protocol was approved by the HESC.

## Author Contributions

AT, EH, KB, and IK, drafted the manuscript and conceptualized the aims and hypotheses. MF-T conducted the analyses. AT and IK set up data collection. All authors provided feedback on different versions of the manuscripts. All authors read and approved the final manuscript and are accountable for all aspects of the work in ensuring that questions related to the accuracy of any part of the work are appropriately investigated.

## Conflict of Interest Statement

The authors declare that the research was conducted in the absence of any commercial or financial relationships that could be construed as a potential conflict of interest.

## Abbreviations

ACT: Acceptance and Commitment Therapy
ANOVA: Analysis of Variance
BISS: Body Image States Scale
BMI: Body Mass Index
DBT: Dialectical Behavior Therapy
EAT-26: Eating Attitudes Test
ED: eating disorder
IPIP: International Personality Item Pool
MB-EAT: Mindfulness-Based Eating Awareness Training
MBCT: Mindfulness-Based Cognitive Therapy
MBSR: Mindfulness-Based Stress Reduction
PANAS: Positive and Negative Affect Schedule
TMS: Toronto Mindfulness Scale
